# Prone versus lateral position in acute hypoxemic respiratory failure patients with HFNO therapy: study protocol for a multicentre randomised controlled open-label trial

**DOI:** 10.1186/s13063-023-07761-8

**Published:** 2023-11-27

**Authors:** Xixin Zhou, Xiaoqing Luo, Qin Li, Guihua Chen, Jin Tong, Wang Deng

**Affiliations:** 1 The Second Clinical College, Chongqing Medical University, Chongqing, China; 2 Department of Pulmonary and Critical Care Medicine, The Second Affiliated Hospital of Chongqing Medical University, Chongqing, China; 3 Nursing Department, The Second Affiliated Hospital of Chongqing Medical University, Chongqing, China

**Keywords:** Respiratory failure, High-flow nasal oxygen therapy, Prone position, Lateral position, Protocol

## Abstract

**Background:**

High-flow nasal oxygen (HFNO) therapy is a leading treatment technique for acute hypoxemic respiratory failure (AHRF), but its treatment failure rate remains high. The awake prone position (APP) has been proven to increase oxygenation and reduce the endotracheal intubation rate in patients with COVID-19-induced AHRF. However, the APP is poorly tolerated in patients, and its performance in improving prognoses is controversial. The lateral position has a similar mechanism and effect to the prone position, but it is more tolerable than the prone position. Therefore, it is worth exploring whether the lateral position is better for awake patients with AHRF.

**Methods:**

This is a protocol for a three-arm parallel-group multicentre randomised controlled open-label exploratory trial. A total of 583 patients from two hospitals in Chongqing, China, will be randomised to take the semi-recumbent position, lateral position, or prone position at a ratio of 1:1:1. Patients are all diagnosed with AHRF secondary to non-COVID-19 pneumonia or lung infection and receiving HFNO therapy. The primary outcome is ventilator-free days in 28 days. The secondary outcomes are the 28-day intubation rate, 28-day all-cause mortality, total position change time, the incidence of adverse events, number of hours using HFNO therapy, length of hospital and intensive care unit (ICU) stay, and others. We will conduct subgroup analyses on the arterial partial pressure of oxygen to the fraction of inspiration oxygen (PaO2/FiO2) ratio (> 200 mmHg or ≤ 200 mmHg), time from admission to intervention implementation (< 24 h or ≥ 24 h), position changing time, and different diagnoses.

**Discussion:**

This trial will explore the prognostic effects of the APP with that of the lateral position in awake patients with non-COVID-19AHRF and compare the differences between them. To provide evidence for clinical decision-making and further research on position management.

**Trial registration:**

This trial was registered in the Chinese Clinical Trial Registry. The registration number is ChiCTR2200055822. Registered on January 20, 2022.

**Supplementary Information:**

The online version contains supplementary material available at 10.1186/s13063-023-07761-8.

## Background

### Background and rationale

Acute hypoxemic respiratory failure (AHRF) is a group of diseases characterised by acute hypoxemia, increased respiratory drive, and respiratory failure, with a mortality of 40% [1–3]. It is the main cause of hospitalisation and mechanical ventilation in the intensive care unit (ICU) [4]. Helmet noninvasive ventilatory support and high-flow nasal oxygen (HFNO) are the most promising, first-line treatment techniques for mild-to-moderate hypoxemia associated with AHRF [5]. And HFNO is more widely used than helmet noninvasive ventilators [6] because it is well tolerated [7], can reduce lung injury [5], and has a lower treatment failure rate compared to standard oxygen therapy [8]. Despite the benefits mentioned above, HFNO’s failure rate is still as high as 48%, of which the intubation rate is 43%, and the 28-day mortality rate is 19% [6]. How to further decrease its treatment failure rate and increase prognoses in AHRF patients with HFNO therapy has become a more significant question for researchers.

The APP is usually considered the prone position applied to awake patients who have not received endotracheal intubation. It was reported to improve oxygenation and decrease endotracheal intubation rate with minimal costs and side effects [9, 10] in awake patients with AHRF secondary to COVID-19. Better effects were reported on patients whose arterial partial pressure of oxygen to the fraction of inspiration oxygen (PaO_2_/FiO_2_) ratio was < 300 mmHg with noninvasive respiratory support [11–15]. And longer duration is associated with better outcomes [3]. However, the APP is poorly tolerated in patients, and there is a lack of an effective method for improving patient tolerance. The mean tolerance time of most patients is less than 3.5 h [3, 16], which is far less than the recommended 12 h [17, 18]. So, detecting the time-effect relationship of the APP is difficult. So far, meta-analyses on APP have not found any other prognosis-related benefits of APP other than decreasing intubation rate [9, 10]. Whether it can improve patients’ prognoses is still controversial [19]. Patient-centred outcomes like ventilator-free days, hospital length of stay, and mortality are all meaningful outcomes. Moreover, ventilator-free days is an outcome that both doctors and patients care about, as using ventilators can be both expensive and cause some serious side effects [5, 20].

The lateral position was proved to have a similar mechanism [21] and effect as the prone position on patients receiving invasive mechanical ventilation [22], and it was easier to tolerate and implement [23, 24]. Researchers placed patients in the lateral position [24, 25] or alternated between the prone position and the lateral position [23, 26] and found that oxygenation was also improved in patients. And previous studies found that the 90° lateral position has the best ventilation effect compared to other angles [27]. However, we have not discovered any experimental study to compare the effects, especially the prognoses, of the lateral position with the prone position on awake AHRF patients [28]. Whether the lateral position is better for awake patients still needs further exploration.

In the preliminary trial, based on feedback from patients and medical staff, we summarized a set of methods to improve patient tolerance. As the COVID-19 pandemic is slowly getting under control, we intend to conduct an experimental study on non-COVID-19 AHRF patients and compare the prognostic effects of the lateral position with the prone position in terms of improved endurance time on them while undergoing HFNO therapy. And to determine whether these positions can help with prognoses and which position is more effective and practical for awake patients. The evidence will help clinicians make better decisions on position management for awake non-COVID-19 AHRF patients undergoing HFNO therapy and set the stage for further research on the effect of APP and that of the lateral position on the prognosis of AHRF caused by various factors.

### Objectives and hypothesis

The first objective of this study was to investigate the effects of prone and lateral positions on patients receiving HFNO therapy in terms of ventilator-free days in 28 days and other prognostic factors. The secondary objective is to compare the differences in the impact of the prone and lateral positions on the investigated factors.

The hypothesis of this study is that positioning AHRF patients in the prone position can lead to improved prognoses. Additionally, for awake patients, the lateral position might be more beneficial than the prone position due to its better tolerance.

## Methods

### Trial design

**Fig. 1:**
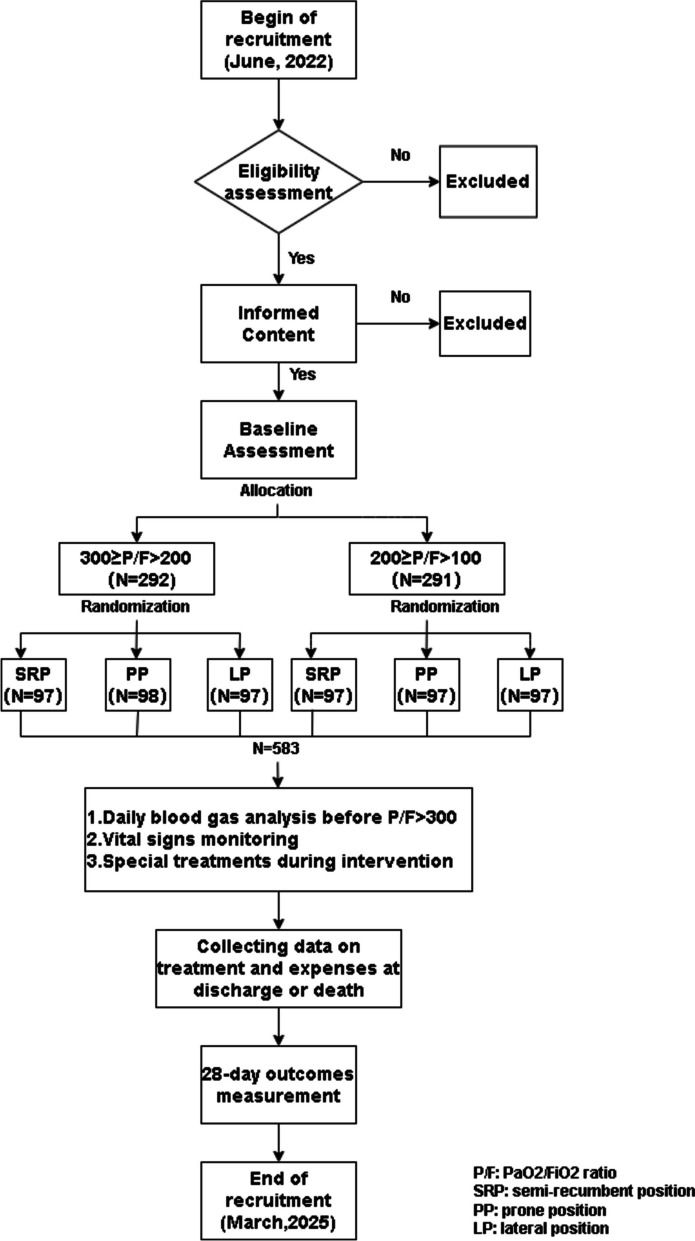
Trial design flowchart. SRP, semi-recumbent position; PP, prone position; LP, lateral position; P/F, PaO_2_/FiO_2_

This is a three-arm, parallel-group, multicentre, randomised controlled, open-label exploratory trial. A total of 583 will be randomly assigned to be placed in the semi-recumbent position (control group), the prone position (experimental Group A), and the lateral position (experimental Group B) at a ratio of 1:1:1. The follow-up period was 28 days. We will conduct and report this trial according to Standard Protocol Items: Recommendation for Interventional Trials. The trial design flowchart is detailed in Fig. 1.

### Study setting

This trial will be conducted in the pulmonary and critical care medicine departments on three campuses of two tertiary A-level teaching hospitals in Chongqing, China, in general wards and the ICUs (The standards for admitting patients to the intensive care unit are shown in Additional file 1). If it is difficult to recruit patients, more departments and hospitals will be involved if they obtain their ethics committee approval.

### Sample size

We calculated the sample size with NCSS statistical software PASS 2021 (version number: 21.0.3). The estimation of ventilator-free days in 28 days for the control group was 19 days, with a standard deviation of 13, based on what was reported before [29] and the severity of the disease [30, 31]. After being placed in the prone position and lateral position, the ventilator-free days in 28 days are expected to increase to 23 days and 22 days, and a standard deviation of 12 and 11 respectively, according to the mean difference in recent researches [10, 29] and the potential benefits of the refined interventions [16, 32–35]. Using an analysis of variance model with a two-sided alpha level of 0.05 and a power of 0.8. The allocation ratio is 1:1:1, so 486 participants are needed. We estimated a dropout rate of 20% due to poor tolerance and our experience in the pilot trial, so a total of 583 patients will be enrolled.

### Recruitment

From June 2022 to March 2025, clinical staff will screen inpatients based on the eligibility criteria. If the patients meet the criteria, trained researchers will recheck and confirm their eligibility. Then inform the patients about the purpose and methods of the study, the risks and benefits of participating, their rights, and the safety and security measures taken during the trial through standardised communication that is easy to understand (Additional file 2). They will then obtain patients’ informed consent (Additional file 3). The patients will then be randomly assigned to three groups and instructed to take their assigned positions as soon as possible.

### Eligibility criteria

#### Inclusion criteria

Adult patients (age ≥ 18 years old) with AHRF, diagnosed with non-COVID-19 pneumonia or lung infection, with PaO2/FiO2 ratios of ˃ 100 mmHg and ≤ 300 mmHg, receiving HFNO therapy with a flow velocity ≥ 30 L/min and FiO_2_ ≥ 30% [36], and giving their informed consent will be included in the study.

#### Exclusion criteria

Patients will be excluded from the study if they:

- Received invasive mechanical ventilation before participating in the experiment during this hospitalisation;
- Need immediate intubation based on a doctor’s assessment;
- Are unable to cooperate or refuse to lie in positions of interest;
- Have haemodynamic instability (Systolic Blood Pressure < 90 mmHg, Mean Blood Pressure < 65 mmHg) or need vasoactive drugs;
- Have deep venous thrombosis or pulmonary embolism;
- Have acute haemorrhagic disease;
- Have thoracic and abdominal trauma or burns, have undergone surgery within three months, or are pregnant;
- Undrained pneumothorax;
- Have increased intracranial pressure or spinal cord injury;
- Terminal-ill patients with a life expectancy of no more than 6 months.

Considering prone position and lateral position may help patients to avoid endotracheal intubation, and patients usually do not decide whether they want to get endotracheal intubation or not until the last moment, so we decided not to exclude patients with “do not intubate” orders.

### Randomisation and concealment

Two investigators who will not participate in this trial will conduct stratified blocked randomisation. First, they will generate random sequences and blocks of various lengths for different centres and layers with IBM SPSS Statistics 25. Then they will obtain grouping results according to the order of the random numbers in different blocks and layers. Subsequently, the grouping results will be placed in opaque sealed envelopes marked with a serial number for the patients’ enrolment order. In addition, they will have a layer mark for the PaO_2_/FiO_2_ ratio category. The envelopes will then be kept by a person who will not participate in the trial at each centre. The person will only open the corresponding envelope when grouping the patients according to their PaO_2_/FiO_2_ ratio (> 200 mmHg or ≤ 200 mmHg) and their enrolment order.

Due to the nature of the interventions, only data collectors and data analysts will be blinded to each participant’s allocation. They will directly extract data from hospitals’ medical and nursing record systems. For unclear information, they will consult trained clinical staff. These staff are asked not to disclose information about patients’ group allocation or the interventions.

### Interventions

We will use Fisher & Paykel Airvo2, Micomme OH-70C, Yuwell HF-75A, Mindray SV300 and other equipment that meets particular requirements for basic safety and essential performance of respiratory high-flow therapy equipment (ISO 80601-2-90:2021). All instruments were provided by the research centres. The researchers in charge of the implementation are nursing team leaders with more than 8 years’ nursing experience. The physicians responsible for disease assessment are doctors with more than 5 years’ experience in ICU diagnosis and treatment.

The parameters of the HFNO therapy system will be set as follows: the initial flow velocity is set at 40–60 L/min. Moreover, FiO2 will be titrated from 30% to maintain pulse oxygen saturation (SpO_2_) at 90%–96%. It will also be dynamically adjusted according to SpO_2_ and blood gas analysis results. If the oxygenation target is not reached, the flow velocity can be gradually increased up to 60 L/min, and FiO_2_ can be increased up to 100%. The temperature ranges from 31℃ to 37 ℃ and will be appropriately adjusted according to each patient’s comfort or tolerance level and sputum viscosity [37].

### Experimental groups

The prone position group (Group A) will take the prone position on a horizontal bed for at least 1 h each time. Patients will be encouraged to take the position as many times as possible each day and remain in the prone position as long as possible each time if they are not tired. The investigators will insert a U-shaped pillow under each patient’s neck or chin and rectangular pillows under their shoulders, chest, hips, knees, and ankles. Then adjust the pillows to ensure their cervical and lumbar vertebrates are in functional positions and patients feel comfortable. Patients’ posture and pillow position can be adjusted at any time with anyone’s help. However, the patients must always keep the abdominal side down and parallel to the bed surface. (More details are provided in Additional file 4).

The lateral group (Group B) will be instructed to alternate lying on each side of their body at an 80°–90° angle on a horizontal bed for at least 1 h each time. Patients with a mono-lateral lung infection will be instructed to lie on the healthy side. Patients will put their upper arms and legs on quilts or pillows placed on the facing side. Their hips will be decompressed to prevent pressure injury. And they will be encouraged to lie on the same side for as long as possible each time and switch to the other side or take the supine position when they feel tired. One round is completed when the patients return to the semi-recumbent position for more than 10 min.

Patients in experimental Groups A and B will be told to change positions at least 1 h after a meal. We track the number of rounds and durations of each round for each assigned position on a daily basis. Only when the patient meets the standards of the corresponding position, and keeps it at least for 1 h at a time, will the duration be considered valid and recorded. Blood gas analysis will be performed daily and 1–2 h after returning to a semi-recumbent position on the first day.

### Control group

Patients in the control group will take the semi-recumbent position (with the bed head elevated 30°–40°) which is the regular position taken by patients. Nurses will routinely instruct the patients to roll over and check vital signs every 2 h. Arterial blood gas will be measured daily. Patients in this group will not take the prone or lateral position unless their doctors think it is necessary.

### Indications for terminating the intervention

1. When the patient’s flow velocity is < 30 L/min and the PaO_2_/FiO_2_ ratio is > 300 mmHg for 6 h [38];
2. The patient can maintain SpO_2_ at 92%–96% [39] with a respiratory rate no more than 25–30 times per minute while using conventional oxygen therapy (nasal prongs, facemask with or without reservoir or Venturi mask with less than 8 litres per minute and the fraction of inspired oxygen less than 35%).
3. The patient’s condition worsens, noninvasive ventilation or endotracheal intubation is needed (see more in “Indications for noninvasive ventilation” and “Indications for endotracheal intubation” section) or death occurs.

### Strategies to improve adherence to the interventions

1. We placed eligible patients in the prone position and conducted a qualitative study on factors affecting the implementation of APP in patients and medical staff. And modified the interventions to make them more acceptable.
2. Researchers explain and encourage patients in a standard way (Additional file 2).
3. We will tell patients the potential benefits they may receive and show them the changes in their PaO_2_/FiO_2_ ratio.
4. Patients’ families will be invited to supervise and encourage patients to take the assigned position.
5. Patients will be asked to take the assigned position continuously without interruption.
6. We will address problems affecting patients’ comfort before taking corresponding positions, such as constipation, intestinal flatulence, and pain.
7. We will place commonly used items (such as water bottles, straws, tissues, phones, urinals, and garbage bins) within patients’ reach.
8. We will ask patients to engage in activities that interest them, such as sleeping and watching videos.

### Withdrawal criteria

1. Patients refuse to undergo HFNO therapy further after enrolment.
2. Patients refuse to change position in the middle of the trial or position change time is <1 h/day.
3. Patients are discharged halfway through the trial.
4. The medical staff thinks that continuing to participate in the study will cause serious harm to the patient.

### Indications for endotracheal intubation

1. Severe respiratory failure does not improve or worsens after noninvasive or high-flow treatment.
2. The patient’s respiratory pattern is seriously abnormal; the frequency is > 40 breaths per minute or < 6–8 breaths per minute; the patient has irregular breathing, weak spontaneous breathing, or disappearance of breath.
3. The patient’s consciousness level decreases, and the Glasgow Coma Scale score is < 10 points.
4. The partial pressure of carbon dioxide increases progressively, and the pH value is <7.25.
5. Airway obstruction that cannot be relieved in the short time.

### Indications for noninvasive ventilation

1. No obvious improvement or aggravation was found in the use of nasal high-flow oxygen therapy (respiratory > 35 times per minute, SpO2 < 88%, paradoxical thoracoabdominal motion, continuous use of adjunctive respiratory muscle)
2. Arterial blood gas analysis showed that PH < 7.35, partial pressure of carbon dioxide > 45 mmHg
3. The ROX index [40] (SpO_2_/FiO_2_ to respiratory rate) < 2.85, < 3.47, < 3.85 after the use of HFNO therapy for 2 h, 6 h, or 12 h respectively, or the above situation occurred within 48 h.
4. The patient cannot maintain SpO_2_ > 90% with FiO_2_ ≥ 60% and a flow velocity of 60 litres per minute via HFNO.

Weaning indications from mechanical ventilation are seen in Additional file 5.

These criteria were all learned and agreed upon by doctors in different centres. The doctors will mainly evaluate whether patients need mechanical ventilation according to the standards above. Investigators will record and evaluate any other reasons for performing mechanical ventilation, as well as cases that meet the criteria but do not get mechanical ventilation.

### Provisions for post-trial care

Routine diagnosis, treatment, and nursing will be determined according to each patient’s condition.

### Outcomes

#### Primary outcomes

The primary outcome variable is ventilator-free days in 28 days. It is defined as days free from invasive mechanical ventilation or noninvasive ventilation within 28 days after randomisation.

#### Secondary outcomes

1. 28-day intubation rate (intubation is defined as endotracheal intubation and receiving invasive mechanical ventilation);
2. 28-day all-cause mortality(defined as death caused by all kinds of diseases within 28 days after randomisation);
3. Change in the daily PaO_2_/FiO_2_ ratio (measured every morning around the same time) during intervention;
4. Change in daily Borg scale score;
5. Daily and total position changing time;
6. Change in daily ROX index: SpO_2_/FiO_2_ to respiratory rate;
7. Number of hours receiving HFNO therapy;
8. Number of hours receiving noninvasive mechanical ventilation and invasive mechanical ventilation;
9. Treatment failure rate (defined as patients who receive noninvasive, invasive ventilation or die after taking assigned positions);
10. The incidence of adverse events;
11. Length of hospital stay and ICU stay.
  Hospitalisation cost.

### Data collection and management

The outcomes will be measured during the intervention and 28 days after the patients are enrolled in the group. The data on the duration of different respiratory support, hospital and ICU lengths of stay, and hospitalisation cost will be collected at discharge or the 28th day after enrolment, whichever occurs first. (The data collection schedule is shown in Table 1). Trained nurses will assess the Borg scale score and fill in the bedside inspection forms (Additional file 6) with average vital signs within 3 min at each allotted timepoint and position change time of each round. Supervisors will examine and transcribe inspection forms every day. Two trained researchers who do not know patients’ allocations will check and collect all other data extracted from the electronic medical record system, including data on withdrawn patients. The main researchers will explain every detail of the parts each researcher is responsible for through online or physical training with a set of standard teaching materials, check the training effect through a test, and give one-to-one guidance to every researcher according to their results.

**Table 1:** Data collection schedule

Study Period	Timepoint	Assessments
Enrolment	Day 0–1	Eligibility
		Informed consent
Allocation		Baseline information
		PaO_2_/FiO_2_ ratio after receiving HFNO therapy for 2 h
Post-allocation	During interventions (Day 1–termination)	Vital signs
		PaO_2_/FiO_2_ ratio
		Borg scale
		Position changing time (experimental groups only)
		ROX index
		Special treatments
		Respiratory support
		Adverse events
	At discharge/Day 28	Hospitalisation cost
		Number of hours using HFNO
		Number of hours using advanced respiratory support mode
		Number of hours using conventional oxygen therapy
		Length of hospital and ICU stay
Close-out	At Day 28	Death
		Endotracheal intubation

The confidentiality of the data will be preserved when the coded, anonymised data are transmitted and stored at the location of the statistician in charge of the final analysis.

### Data monitoring

The data monitoring committee will be composed of a nurse, a doctor, and a statistician independent of the investigators and without competing interests. They will check data integrity, accuracy, authenticity, and timeliness daily. They will make suggestions to ensure data quality and suggest stopping the trial if safety issues arise.

### Adverse outcomes and precautions

The probabilities of adverse events, including pressure injury, arrhythmia, asphyxia, and unplanned extubation (the definitions are seen in Additional file 7), have been reported to be small [16].

By fully educating patients with a teaching video before the start of the trial, we will instruct them to take a standard lying position and teach them how to adjust their positions and decompress pillows to make them feel comfortable and avoid pressure injuries. All kinds of tubes will be properly fixed and secured with the investigators’ help. Patients will be told to sit up or take the supine position and ring the bell to call the nurse immediately in case of palpitation, chest tightness, or dyspnea to stop the development of threats.

Although the probability of fatal adverse events occurring is low, there is still a risk of cardiac arrest [3]. Observations by clinicians indicate that patients’ intolerance and cardiac discomfort generally occur within the first hour of treatment. To ensure patients’ safety, for the participants in the experimental groups (Group A and Group B), the patients’ vital signs will be consistently monitored; the investigators will check each patient at the 15-min, 30-min, and 1-h time points, and then every 1 h using a standardised inspection checklist (Additional file 5). The participants’ vital signs in the control group will be checked every 2 h. We encourage nurses to use special symbols that their leaders do not know to represent the event when adverse outcomes happen, to increase reporting behaviour.

### Descriptive analysis

The baseline characteristics will be presented based on different layers of the PaO_2_/FiO_2_ ratio. Quantitative variables will be described using the mean ± standard deviation (M ± SD) or the median quartile interval (median, IQR). Categorical variables will be described in percentages. The significance level was set at 0.05.

### Statistical methods for the primary and secondary outcomes

Analysis of variance will be used for ventilator-free days in 28 days, the number of hours using HFNO, noninvasive ventilation, invasive mechanical ventilation, length of hospitalisation, and hospitalisation cost. When it indicates that at least one group is significant, the Bonferroni correction will be applied.

For the 28-day mortality rate and 28-day intubation rate, we will conduct a survival analysis with the Kaplan-Meier method. We will compare the mortality and intubation rates of the three study groups using a log-rank test. We will use the Cox proportional hazards model to detect the relationship between diagnosis, onset time, PaO_2_/FiO_2_ ratio change, the duration of changing positions, and total position changing time.

Other secondary outcomes, such as the change in the PaO_2_/FiO_2_ ratio, ROX index, Borg scale score within the groups, will be compared with a paired repeated measures analysis of variance or generalised estimating equation based on the integrity of the data. The incidence of adverse events will be analysed using either the chi-square test or Fisher’s exact test. Univariate and multivariate analyses of risk factors for prone position failure will be performed with logistic regression.

### Methods for additional analyses

We will conduct subgroup analyses based on the PaO_2_/FiO_2_ ratio (PaO_2_/FiO_2_ > 200 mmHg or ≤ 200 mmHg), time from admission to intervention implementation (< 24 h or ≥ 24 h), position changing time, and different diagnoses. The Bonferroni correction will be used for pairwise comparisons among the three groups. We will handle the missing values of withdrawn patients with the intention-to-treat the analysis and use multiple imputation. We will also do a per-protocol analysis if patients in the supine position group take the intervention that the experimental groups have. Since this is a multicentre study, centre-effect analysis will be implemented.

### Patient and public involvement statement

Patients’ opinions and feedback on the duration of changing positions and the measurement to improve comfort were adopted and helped us to revise the protocol.

## Discussion

Ehrmann and colleagues started the world’s largest randomised controlled trial on APP so far and proved it could improve oxygenation and avoid endotracheal intubation in patients with COVID-19-induced AHRF with few costs and complications [3]. Although another nonrandomised controlled trial showed APP has no clinical benefit and has potential harm among patients [41], it was thought to be caused by a baseline imbalance between groups [19]. However, what has not been proven is whether APP can increase patients’ prognoses, such as ventilator-free days, hospital length of stay, and mortality. Ehrmann and colleagues suggested that a duration of APP no less than 8 h was associated with greater treatment success, while their reported median daily duration was 5 h, and showed no big difference (median difference ± 0.2) in the mean duration of invasive mechanical ventilation, hospital length of stay, or mortality between the APP group and the standard group [3]. The Mexican centre of this trial had a median daily prone position duration of 8.6 h and had a larger difference on days of intensive mechanical ventilation and hospital length of stay (median difference −1.5, *P* < 0.001). We inferred that a median daily APP duration of no less than 8 h can help reduce AHRF patients’ prognoses. But the reason for the longer duration was reported as consistent encouragement and in-person assistance on a 24/7 basis [32], which is not practical for clinical occasions. And there is a lack of more practical and effective skills to improve patients’ tolerance to APP, which highly affects the exploration of the time-effect relationship on patients’ prognoses. Its performance in improving prognosis is controversial [19].

The lateral position was reported to be better tolerated and had effects similar to the prone position in intubated patients [42]. However, the current experimental studies on the APP only compare the prone position with the semi-recumbent position. Most lateral supine position studies were case reports or combined prone and lateral supine positions. There is a lack of experimental evidence comparing the lateral position and the APP. Which one has a better effect on patients is unknown. Moreover, as the COVID-19 pandemic is slowly under control, it is more practical to switch attention to non-COVID-19 AHRF, which has a similar mortality rate to COVID-19-induced AHRF [30, 31]. And previous observational studies have shown that APP is beneficial to non-COVID-19 patients [11]. So, we plan to conduct a multi-centre randomised controlled trial on patients with non-COVID-19 AHRF in the APP and the lateral position compared with the standard semi-recumbent position to explore the prognostic effects of those positions on patients at maximum tolerating time and their differences.

This protocol has several advantages. First, it compares the APP with the lateral position in patients with non-COVID-19 AHRF in a randomised controlled trial that we have not seen in other studies. Second, we worked out measures to improve patient tolerance practically based on investigating the factors that affect patients and medical staff implementing the APP with a qualitative study we did before. It is intended to maximise the function of the APP, so we can compare it with the lateral position using the data obtained in actual clinical situations. Third, we provide details on improving patients’ tolerance to the APP, so more researchers can refer to it when studying and implementing the APP. Fourth, as we chose the APP, the lateral position, and the semi-recumbent position, we can provide three aspects of evidence for exploring the prognostic effects of the interested positions on patients with non-COVID-19 AHRF receiving HFNO therapy.

However, our design has the following limitations. First, due to the nature of the intervention, the patients and medical staff involved in the trial were not blinded to the allocation. Although the investigators who collected the data were blinded to the allocation, there was still a chance of exaggerating the intervention effects. Second, the patients are from the same area, so there is a lack of representation and universality. Third, a relatively short follow-up period could affect a full exploration of the prognosis.

In summary, this research aims to provide evidence on the prognostic effects of the prone position and the lateral position on patients receiving HFNO therapy and their differences to help clinicians make better decisions on position management and provide a basis for further research.

## Trial status

This trial is in the enrolment stage. The protocol is the fourth version and was revised on October 2022. The recruitment began on June 1, 2022, and is expected to be completed in March 2025.

### Supplementary Information

**Additional file 1. **The standards for admitting patients to the ICU.

**Additional file 2.** Standard communication procedure.

**Additional file 3.** Informed consent.

**Additional file 4.** Operating checklist of APP.

**Additional file 5.** The weaning indications for mechanical ventilation.

**Additional file 6.** Inspection checklist.

**Additional file 7.** Definitions of adverse outcomes.

## Data Availability

The datasets used and/or analysed during the current study are available from the corresponding author upon reasonable request.

## Abbreviations

HFNO: High-flow nasal oxygen
AHRF: Acute hypoxemic respiratory failure
APP: Awake prone position
ICU: Intensive care unit
PaO_2_/FiO_2_: The arterial partial pressure of oxygen to the fraction of inspiration oxygen
SpO_2_: Saturation of peripheral oxygen
M ± SD: Mean ± standard deviation
Median, IQR: Median, interquartile range
