# Supplementary material for: Prone versus lateral position in acute hypoxemic respiratory failure patients with HFNO therapy: study protocol for a multicentre randomised controlled open-label trial
Source: Trials. 2023 Nov 27;24:762. doi: 10.1186/s13063-023-07761-8 (PMC10683165; doi:10.1186/s13063-023-07761-8)
Supplement: Supplementary file 1 — Additional file 1. The standards for admitting patients to the ICU. [file 13063_2023_7761_MOESM1_ESM.pdf]

The standards for admitting patients to the intensive care unit:

1. Patients with acute respiratory distress syndrome or severe pneumonia with respiratory failure undergoing mechanical ventilation treatment or septic shock who need to closely monitor their vital signs

2. Patients with multiple organ dysfunction or unstable vital signs after whole lung lavage surgery that require close monitoring

3. Patients with acute attacks of chronic obstructive pulmonary disease or critical asthma and respiratory failure who need invasive ventilation

4. Patients with acute massive pulmonary embolism and hemoptysis who need to be rescued at any time

5. Other critically ill patients may undergo changes in their condition at any time and require monitoring and rescue.
