# Supplementary material for: Prone versus lateral position in acute hypoxemic respiratory failure patients with HFNO therapy: study protocol for a multicentre randomised controlled open-label trial
Source: Trials. 2023 Nov 27;24:762. doi: 10.1186/s13063-023-07761-8 (PMC10683165; doi:10.1186/s13063-023-07761-8)
Supplement: Supplementary file 2 — Additional file 2. Standard communication procedure. [file 13063_2023_7761_MOESM2_ESM.pdf]

Standard communication

Content

Standard communication during enrollment phase.................................1

Standard communication during implementation phase 5

Explain before operation..5

Explain during operation 5

Explanation after operation 6

Patient Refusal Communication Process 7

Before operation..7

During operation 8

After operation 8

Standard communication process..10

for lateral position 10

Explanation before action 10

Explanation during operation..10

Explanation after operation 10

Patient Refusal Communication Process 12

Before operation 12

During operation 13

After operation 13

Standard communication during enrolment phase

Hello, sir/mam. I am a member of the medical staff in this department. My name is ***. (Show them the work card.) We are conducting a study about the effects that different positions have on patients using high-flow nasal oxygen therapy, and you meet the conditions for participation without any contraindication. We would like to invite you to join this study. If you join this study, the interventions may help you to relieve your symptoms, reduce the possibility of endotracheal intubation and even help you avoid further development of the disease, recover faster, and be discharged quicklier.

If you agree to join this study, you will be randomly assigned to one of our research groups. Then trained experienced nurses will guide you to take the relevant lying position and make you feel comfortable. And you need to strictly obey our instructions and try to keep the required position as long as possible each time. According to your group and your health situation, nurses will take your arterial blood 1-3 times a day to check your status, based on the doctor’s judgment. The position you will take has not previously reported any serious complications and was tested by our staff to ensure safety before applying it to you. When you take this position, we will closely monitor you, and investigators will visit hourly to ensure your safety. We will use pillows to protect your skin from being compressed. Once you feel palpitations, chest tightness, or dyspnea, please immediately sit up and call us; we will check on you immediately.

You have the right to exit the trial whenever you want, and we will suspend your test when your doctor thinks you are not fit to continue the practice. If you have irreversible damage due to our intervention, you will be compensated by our department. Your personal treatment-related

information and data will only be used for diagnosis, treatment, nursing care, and this research, and we will not expose your privacy. Your blood is only for testing; it will be discarded after being used, not for other use.

Standard communication for awake prone position

Explanation before operation

Hello sir/madam, Because you have an infection in your lungs, your fatigue and panting are evident. Now your doctor has prescribed prone ventilation for you, which is to let you lie on your stomach. The purpose is to reduce your wheezing symptoms and help you recover faster. There are many patients with similar conditions to you lying on their stomachs, and we can see that the index of his hypoxia (SpO_2) improves quickly (within a few minutes). This operation is simple and free of charge and does not add a financial burden to you, but it requires your cooperation and persistence. We are here to guide you and help you better master and implement this skill. Do you think it is convenient for you now? Have you been coughing up blood recently? (If so, ask for color, quantity, and character, and judge whether it is acute massive bleeding.) Have you eaten in the last hour? Do you need to go to the bathroom?

Explanation during operation

Ask the patient if there is any discomfort, and solve it in time.

If you cannot lie completely supine, we can raise the head of the bed by 10°–20°.

If you feel that the U-shaped pillow is strangling the neck, put the U-shaped pillow down and put the chin on the U-shaped pillow or other pillows to keep the head and back level (to ensure that the neck does not sage or fall back).

If you feel pressure on your chest or abdomen, move the pillow up or down to let that part hang in the air.

If you feel bored, let the patient play with their mobile phone, watch TV or video, listen to music, etc. to divert attention.

Explanation after operation

In order to achieve better results, experts recommend that the longer the prone time, the better, and the current recommended time ranges from 6 to 12 hours. This is difficult to achieve at one time, but it can be divided into multiple times to get closer to this goal. If you don't feel uncomfortable, try to take the prone position a little longer at a time and do it a few more times. If you are not used to it, it does not matter. We set a minimum goal of at least one hour each time, try to increase the number of times you take the prone position, and then slowly increase the time after you get used to it. Don't be stressed. We will just try our best to achieve it for the longest time we can. This is better than doing nothing. If you have any discomfort in the middle, please let us know in time, and we will solve it for you immediately.

Patient Refusal Communication Process

Ask the patient: Do you have any concerns? (To give targeted explanations later)

Before operation

Doubt effect and authenticity:

During the COVID-19 period, including now, many places and many patients are using this method. It is simple, fast and effective. If you don't believe it, you can check it. It is recommended in the guidelines issued by the state. Of course, this may not be effective for everyone, it is mainly related to diseases and individuals. In theory, you are eligible for the prone position, mainly depending on your willingness and cooperation. But so many people like you use it effectively without spending money, even if it doesn't work for you, there is not much loss. But if the symptoms are relieved, it will not be far from being discharged from the hospital. You and your money get will benefit from it, so why not trying it?

Feel troublesome:

In fact, this operation itself is not troublesome, just lie on the stomach. After a few time, many patients will be able to do it themselves. But it's a matter of persistence. If you don't think you will persist, we will supervise and urge you. Now we all advocate rapid recovery, and your speedy recovery is also our wish and goal. We work together to get you discharge from the hospital as soon as possible. If you like to sleep on your stomach yourself, you can even do it when you are sleeping. If you don't , then just

regard it as a simple and easy way to get treatment.

During operation

The patient refuses to cushion the pillow

The pillow is to reduce the pressure on the chest and abdomen and, at the same time, reduce the problem of neck and waist pain after lying for a long time, so that you can be more comfortable, persist for a longer time, and the effect will possibly be better. If you feel that the pillow's position is not good in the middle, you can move your body to adjust it yourself, or ask us or your family to help you adjust the position of the pillow.

The patient feels too hot

We have cooling stickers, water pads and small fans for you to use to help you cool down. Try it first to see if it will be better. (If the patient still refuses, let the patient have a rest first and choose a time when the patient feels comfortable (not hot).)

After operation

Feel uncomfortable

Where do you feel uncomfortable? It's normal for you to feel uncomfortable, probably because...(the reason) we will change it to.. It should be solved. Will that be ok for you?

If the patient does not agree with the improvement measures; how do you think the changes are more acceptable to you? (Discuss with the patient and choose an improvement plan that the patient can accept.)

Don't think it is useful

If the patient's vital signs improve

Let’s see, this indicator before you took the prone position was..., After you took the prone position was.. (It is best to let the patient see for himself; by wearing a portable finger pulse oxygen monitor, the patient can see the changes and contrasts in real time, so as to enhance the patient's confidence in the prone position.) There is improvement, but for short-term changes, you may not feel it yourself. Let's do it a few more times, and over time, you'll be able to feel it slowly.

If the patient's vital signs do not improve significantly

This may be related to the short time you lay or the late start time. Because you don't have any discomfort after lying down, we will try to take the prone position for a longer time next time to see if it will be better after the accumulated time is longer. Because experts suggest that the time is still very long after all. Will that be ok for you?

Standard communication process for the lateral position

Explanation before action

Hello sir/madam, Because you have an infection in your lungs, your fatigue and panting are evident, and taking a supine position all the time is not conducive to sputum discharge and lung anti-inflammatory. Sleeping on your side can help you use gravity to draw out the phlegm. This will reduce inflammation faster, breathe smoother, and avoid snoring, so you can get better faster. Your doctor asks you to sleep on your side. Do you think it is convenient for you now? Have you coughed blood recently? (If so, ask for colour, quantity, and character, and judge whether it is acute and massive bleeding.) Have you eaten in the last hour? Do you need to go to the bathroom?

Explanation during operation

(Ask the patient if there is any discomfort, and address it as soon as possible.)

If you cannot lie completely supine, I can raise the bed head by 10°–20°.

If you feel pain in your hip, we can use a pressure relief tool such as a pillow or a pressure relief patch to reduce pressure.

If you feel bored, you could play on the mobile phone, watch TV or video, listen to music, etc., to divert your attention so you can feel time fly faster.

Explanation after operation

In order to achieve better results, please spend more than an hour on each side, and try to sustain yourself as long as possible each time. If you feel unbearable, sleep on the other side. And the same for the other side. Try to keep it for more than an hour. If there is no discomfort, try to last as long as possible each time. If you feel unbearable, sleep on the other side. Alternate on both sides.

In addition to having meals, atomization, going to the toilet and other things that must be done, try to sleep on your side at other times. The longer the time to sleep on your side, the better.

Please let us know if you feel any discomfort in the middle, and we will fix it.

Patient Refusal Communication Process

Ask the patient: Do you have any concerns? (To give targeted explanations later.)

Before operation

I have no phlegm

The auscultation assesses whether the patient really has no phlegm sounds.

If there is—you just have phlegm, the phlegm is too thick, or you have no strength to cough up, we can better help to remove phlegm through treatment, combined with the lateral position.

If there is not—the lateral position can not only help expectoration but also reduce the impact of gravity on a specific position in the lungs, promote the absorption of inflammation, and help you recover. It is very effective for some patients to use the side position during COVID-19, but it is more effective to use the side position for a long time (6–12 hours), so we encourage you to sleep on the side for as long as possible.

Doubt effect and authenticity

We have a method to help expectoration called posture drainage, which is achieved by changing posture. This is written in the textbook, and you can check it online. At the same time, it is very effective for some patients to use the side position during COVID-19, but it is more effective to lie on the side for a long time (6–12 hours), so we encourage you to sleep on the side for as long as possible.

Feel troublesome

In fact, this operation itself is not troublesome; just lie sideways. But it's a matter of persistence. If you can't persist, we will supervise and guide you. As we all advocate rapid recovery, your speedy recovery is our wish and goal as well. We will work together to get you discharged as soon as possible. If you like to lie sideways, you can even do it when sleeping. If you don't usually sleep sideways, just regard it as a treatment simply by changing a position.

During operation

The patient cannot lie supine

If you really can't lie on your back, I can also raise the bed head a little higher (10°-20°).(If it still doesn't work, raise the bed head to a height that the patient can bear. And note in the data collection form.)

Pain in the compressed area when the patient lies on his/her side

I will give you a pad or pillow to decompress you, and you will feel better. (Depending on the patient's economic level and what they already have, such as water cushions, folded quilt covers, small pillows or decompression pads for protection.)

After operation

Feel uncomfortable

Where do you feel uncomfortable? It's normal for you to feel uncomfortable, probably because……(the reason).We will change it to...

(solutions to it), it should be solved, will that be OK?

If the patient does not agree with the improvement measures

How do you think the changes are more acceptable to you? (Discuss with the patient and choose an improvement plan that the patient can accept.)

Don't think it works

If the patient's vital signs improve

Please look at this; before you lie sideways, this indicator was... After you lay sideways, (It is best to let the patient see for himself or herself; by wearing a portable finger pulse oxygen monitor, the patient can see the changes and contrasts in real time, to enhance the patient's confidence in the prone position.) There is improvement.

But for a short time, you may not feel it yourself. Let's do it a few more times, and over time, you'll feel it slowly.

If the patient's vital signs do not improve significantly

The short time you spent lying sideways or the late start may have caused this. Because you have no discomfort after lying down, we can try to lay down for a longer time next time. This is to see if it will be better after the period is longer. Experts still suggest a very long time after all. Will that be OK for you?

If the patient coughs more sputum

You have more sputum when you lie on your side, which is good. When the sputum is removed, you will recover faster.
