# Supplementary material for: Prone versus lateral position in acute hypoxemic respiratory failure patients with HFNO therapy: study protocol for a multicentre randomised controlled open-label trial
Source: Trials. 2023 Nov 27;24:762. doi: 10.1186/s13063-023-07761-8 (PMC10683165; doi:10.1186/s13063-023-07761-8)
Supplement: Supplementary file 3 — Additional file 3. Informed consent. [file 13063_2023_7761_MOESM3_ESM.pdf]

Informed Consent

Research Background:

You will be invited to participate in a study conducted by Guihua Chen (86-13637889425) and the Department of Respiratory and Critical Care Medicine, the Second Affiliated Hospital of Chongqing Medical University. This is a study to demonstrate whether different supine ventilation improves disease outcomes in patients treated with nasal high-flow oxygen therapy, and it will last 12-24 months. You were invited to join this study because you are on nasal high-flow oxygen therapy and have been evaluated by your doctor as no contraindications.

This informed consent form provides you with information to help you decide whether to participate in this clinical study. Your participation in this study is voluntary. The study has been reviewed by the Institute's Ethics Review Committee. If you agree to join this study, please see the instructions below. Please read it carefully and ask the investigator in charge of the study if you have any questions.

Objectives of the study:

Corresponding ventilation refers to a form of breathing that takes place over a long period of time in the corresponding position, which has been shown to reduce mortality in intubated patients and is often used in intubated patients who sedative and analgesic.

The 2016 China Mechanical Ventilation Guidelines recommend that patients with severe acute respiratory distress syndrome (ARDS) should be mechanically ventilated in corresponding positions.

During the new crown pneumonia epidemic, a large number of foreign medical institutions used corresponding ventilation in patients with coronavirus using non-invasive ventilators or other non-intubation, and the results suggested that most patients had significant improvement in symptoms, and corresponding ventilation may help patients avoid endotracheal intubation and there are few adverse reactions reported.

However, there is little evidence to confirm whether postural changes can last for symptom improvement, how long corresponding is more effective, whether corresponding ventilation can reduce or avoid intubation in patients using noninvasive ventilators, and whether patient mortality can be reduced.

Therefore, this trial intends to verify whether corresponding ventilation can help patients using nasal high-flow oxygen therapy avoid endotracheal intubation and reduce mortality. At the same time, explore which of the corresponding and lateral positions is more effective and more suitable for clinical patients.

Research process and methods:

The study will conduct a one-month pre-trial in January to study the time and frequency of patient corresponding falls, calculate the average corresponding time, explore the factors that affect the length of patient corresponding lying, and improve the process. Patients who had undergone high-flow oxygen therapy through the nose were interviewed about how they felt when they were in the relevant recumbent position, and whether they felt that their symptoms had improved. Interviews were conducted with obligated persons who had participated in the non-invasive corresponding position, including difficulties in implementation and difficulties. Targeted improvement programs are made for the issues raised by patients and medical staff.

After the formal trial, patients will be positioned according to randomized groups, the high-flow nasal oxygen humidification instrument will be adjusted, first-level care will be given, and the arterial blood sample will be sampled on time and examined as planned. At the same time, the patient's corresponding time, frequency, blood gas analysis, vital signs, ventilator parameters, days of use of the ventilator, intubation and death are recorded. The trial will select 150 eligible candidates from two top three hospitals in Chongqing for randomization and comparative study.

If you agree to participate in this study, we will number each subject and establish a medical record file. You will be randomly grouped in the course of the study, during the study and we will take arterial blood to do blood gas analysis according to your condition and study plan, which was treated immediately after the arterial blood test was completed and not used for other purposes.

Possible benefits of the study:

By changing the recumbent position in combination with high nasal flow, it may improve your symptoms of hypoxia, promote recovery from illness, avoid endotracheal intubation, shorten the length of hospital stay, and reduce the cost of your hospital stay.

Research risks and discomfort:

Your sample collection will be performed strictly as sterile and there may be some very small risks during specimen collection, including transient pain, local bruising, mild dizziness in a small number, or an extremely rare needle infection.

When taking those positions, you may have tenderness and redness in the body. At this point you can inform your medical staff and change your position appropriately to reduce discomfort, and if necessary, we will use pillows for you to relieve local stress and promote your comfort to help you continue to perform corresponding surgery. A small number of patients may also have heart discomfort symptoms such as palpitation and chest tightness, at which point we will deal with the condition according to your symptoms, vital signs, your subjective wishes and the doctor in charge, and determine whether you are suitable for continuing the relevant recumbent ventilation.

During the entire process of your corresponding lying, there will be medical staff at the bedside or rush to the bedside in time after your call, and the doctor who will make the

judgment of your condition is the doctor in charge who understands your condition. Your care is attended by a nurse with two years or more of experience in the respiratory intensive care unit.
Privacy issues: (Privacy protection in the process and privacy protection in the results of publication).

If you decide to participate in this study, your participation in the trial and your personal data during the trial are confidential. Your test results will be placed in the medical record holder and will be managed according to the standards of medical record management regulations. Your personal information and medical information will not be available to you without your authorization or by the nursing staff at this time. Your personal information will not be covered when the results of this study are published.

Fees and Compensation:

If you are involved in an irreversible injury related to that clinical study as a result of your participation in this study, you may receive free treatment and/or corresponding compensation. /b10>The cost of treatment is provided by the study.

Free Exit:

As a subject, you can stay informed of the information and progress of the study and voluntarily decide whether to (continue) or not to participate. After participating, regardless of whether the injury has occurred, or whether it is serious, you may choose to notify the investigator at any time to request withdrawal from the study, your data will not be included in the results of the study, and any of your medical treatment and rights will not be affected. If you continue to participate in the study, it will cause you serious harm and the researcher will also suspend the study.

However, during your participation in the study, please provide the true facts about your medical history and current physical condition; tell the research doctor about any discomfort you have developed during this study; do not take restricted drugs, food, etc.; and tell the research doctor whether you have recently participated in other studies or are currently participating in other studies. If you are not complying with the research program, or if a research-related injury has occurred or for any other reason, the research physician may terminate your continued participation in the study.

Contact:

If you have questions related to this study, or any discomfort and injury you have incurred during the course of your study, or have questions about the rights and interests of participants in this study, you can contact **Guihua Chen** at **86-13637889425.**

Post-trial benefit sharing:

If the study is completed and the patient who changes the recumbent position for high-flow nasal oxygen therapy can improve symptoms and promote patient improvement, we will further summarize this experience and promote it comprehensively within the discipline.

Informed consent signature:

I have read this informed consent form, and my doctor_(signatory) has explained to me in detail the purpose, content, risks, and benefits of this clinical trial, and has answered all the questions I asked, I am aware of this clinical study, and I have volunteered to participate in this study.

Patient’s Signature:__________________________

Date: ______ _Year_________________
