# Supplementary material for: Prone versus lateral position in acute hypoxemic respiratory failure patients with HFNO therapy: study protocol for a multicentre randomised controlled open-label trial
Source: Trials. 2023 Nov 27;24:762. doi: 10.1186/s13063-023-07761-8 (PMC10683165; doi:10.1186/s13063-023-07761-8)
Supplement: Supplementary file 4 — Additional file 4. Operating checklist of APP. [file 13063_2023_7761_MOESM4_ESM.pdf]

Operating Checklist of The Awake Prone Position

Operation		Date									
Before	1h after a meal										
	no interruption factor wiin 1h										
	U-shaped pillow + pillow ⩾ 4										
	No other discomfort (defecation, abdominal distension, pain, cough,etc)										
	All tubes are well-fixed										
	Leave enough room for lines										
During	Move to the right edge of the bed (hip, shoulder, neck)										
	Wear a U-shaped pillow hackward on the neck										
	Prepare pillows (parallel to shoulder, iliac, knee)										
	Bend your right leg and stand on the bed										
	Stretch your arms over the top of your head										
	Push the right leg to turnover										

重医二院PCCM

Operating Checklist of The Awake Prone Position

	Adjust the body's / pillow's position										
	Adjust tube's / line's location										
After	Cervical and thoracic vertebrae at the same lecvel										
	Lumbar and thoracic vertebrae at the same level (for those who get backache easily										
	Tubes are not fold										
	Tubes don't press skin										
	No oppressive discomfort										

重医二院PCCM
