# Supplementary material for: Prone versus lateral position in acute hypoxemic respiratory failure patients with HFNO therapy: study protocol for a multicentre randomised controlled open-label trial
Source: Trials. 2023 Nov 27;24:762. doi: 10.1186/s13063-023-07761-8 (PMC10683165; doi:10.1186/s13063-023-07761-8)
Supplement: Supplementary file 5 — Additional file 5. The weaning indications for mechanical ventilation. [file 13063_2023_7761_MOESM5_ESM.pdf]

The weaning indications for mechanical ventilation

Invasive mechanical ventilation

1. Basic diseases are under control: the basic diseases that induce breathing difficulties are under control, the condition tends to stabilize, and the symptoms tend to alleviate;

2. Physiological indicators standard: Before removing the ventilator, observe the patient's physiological indicators, such as oxygenation >150-200mmHg; Positive end expiratory pressure $\leqslant$ 5-8cmH_2O; Inhaled oxygen concentration $\leqslant$ 40% -50%; Arterial blood pH $\geqslant$ 7.25; Chronic obstructive pulmonary disease, with arterial blood pH>7.30, arterial partial pressure of oxygen>50mmHg, and inhaled oxygen concentration<0.35, indicates that the indicators return to normal;

3. Hemodynamics is stable: there is no dynamic change of myocardial ischemia, there is no significant hypotension clinically, and vasoactive drugs are not needed or only small doses of vasoactive drugs, such as dobutamine hydrochloride, are needed for infusion;

4. Respiratory function improvement: At this point, there is the ability to breathe autonomously and have good airway protection. Coughing is powerful, and secretions are significantly reduced.

Non-invasive ventilation

1. A respiratory rate between 12 and 22 breaths per minute.
2. Peripheral oxygen saturation (SpO_2) ⩾90 percent on ⩽60 percent FiO2 or predicted needs can be met with oxygen delivered via high-flow nasal cannula (HFNC) or low-flow oxygen.
3. Hemodynamic stability (preferably off or on low-dose vasopressors and heart rate ⩾50 and ⩽120 beats per minute).
4. The pH is preferably >7.25, and the patient should ideally be afebrile, awake and alert, or easily arousable.
5. Minimal NIV settings (e.g., bilevel positive airway pressure of 10 cmH_2O)

The doctors will mainly evaluate whether patients need to wean from mechanical ventilation according to the standards above. Investigators will record and evaluate any other reasons for weaning from mechanical ventilation, as well as cases that meet the criteria but do not wean from mechanical ventilation.
