# Supplementary material for: Prone versus lateral position in acute hypoxemic respiratory failure patients with HFNO therapy: study protocol for a multicentre randomised controlled open-label trial
Source: Trials. 2023 Nov 27;24:762. doi: 10.1186/s13063-023-07761-8 (PMC10683165; doi:10.1186/s13063-023-07761-8)
Supplement: Supplementary file 6 — Additional file 6. Inspection checklist. [file 13063_2023_7761_MOESM6_ESM.pdf]

Prone position inspection checklist

Bed number			Name		Admission number			Date	Time	
Assessment / Time	FiO2	Flow	Pulse	Respiratory rate	Blood pressure	SpO2	Borg scale	Tubes got fold or compress-ed	Area of discomfort and symptoms	Rox index
Before internvention										
15min										
30min										
1h										
2h										
3h										
4h										
5h										
6h										
7h										
8h										
Duration of this round:										

Discomfort includes:discomfort in eye, neck, chest, costal margin, hands, abdomen, iliac spine, back, knee, ankle and toe, reflux, hot, etc

Cution: When the neck and waist are sore or painful, the pillows' height and positions must be adjusted, so that the patient's cervical spine and lumbar spine are in their functional position.

Bed number			Name		Admission number			Date	Time	
Assessment / Time	FiO2	Flow	Pulse	Respiratory rate	Blood pressure	SpO2	Borg scale	Tubes got fold or compress-ed	Area of discomfort and symptoms	Rox index
Before internvention										
15min										
30min										
1h										
2h										
3h										
4h										
5h										
6h										
7h										
8h										
Duration of this round:										

Discomfort includes:discomfort in eye, neck, chest, costal margin, hands, abdomen, iliac spine, back, knee, ankle and toe, reflux, hot, etc

Cution: When the neck and waist are sore or painful, the pillows' height and positions must be adjusted, so that the patient's cervical spine and lumbar spine are in their functional position.

The lateral position inspection checklist

Bed number		Name		Admission number			Date	Time	
Assessment Time	FiO2	Flow	Pulse	Respiratory rate	SpO2	Borg scale	Tubes got fold or compressed	Area of discomfort and symptoms	Rox index
Before internvention									
15min									
30min									
1h									
2h									
3h									
4h									
5h									
6h									
7h									
8h									
Duration of this round:				the left side duration			the right side duration		

Discomfort includes:discomfort in eye, neck, chest, costal margin, hands, abdomen, iliac spine, back, knee, ankle and toe, reflux, hot, etc

Cution: When the neck and waist are sore or painful, the pillows' height and positions must be adjusted, so that the patient's cervical spine and lumbar spine are in their functional position

Bed number		Name		Admission number			Date	Time	
Assessment Time	FiO2	Flow	Pulse	Respiratory rate	SpO2	Borg scale	Tubes got fold or compressed	Area of discomfort and symptoms	Rox index
Before internvention									
15min									
30min									
1h									
2h									
3h									
4h									
5h									
6h									
7h									
8h									
Duration of this round:				the left side duration			the right side duration		

Discomfort includes:discomfort in eye, neck, chest, costal margin, hands, abdomen, iliac spine, back, knee, ankle and toe, reflux, hot, etc

Cution: When the neck and waist are sore or painful, the pillows' height and positions must be adjusted, so that the patient's cervical spine and lumbar spine are in their functional position
