# Supplementary material for: Prone versus lateral position in acute hypoxemic respiratory failure patients with HFNO therapy: study protocol for a multicentre randomised controlled open-label trial
Source: Trials. 2023 Nov 27;24:762. doi: 10.1186/s13063-023-07761-8 (PMC10683165; doi:10.1186/s13063-023-07761-8)
Supplement: Supplementary file 7 — Additional file 7. Definitions of adverse outcomes. [file 13063_2023_7761_MOESM7_ESM.pdf]

Definitions of adverse outcomes

Adverse outcomes and Countermeasure

1. Pressure injury: to define according to the International Guideline of Prevention and Treatment of Pressure Ulcers/Injuries
2. Arrhythmia: caused by abnormal excitation of sinoatrial node or produced outside sinoatrial node, and the conduction of excitement is slow, blocked or transmitted through abnormal channels, that is, the origin of cardiac activity and / or conduction disturbance lead to abnormal frequency and / or rhythm of heart beat. should be diagnosed by ECG result.
3. Asphyxia: caused by sputum blockage: patients with sudden decrease in oxygen saturation, dyspnea, need emergency sputum suction or bronohoalveolarlavage, or even endotracheal intubation.
4. Unplanned extubation: accidental loss of catheter or without the consent of medical staff.
